# A global systematic review and meta-analysis on laparoscopic vs open right hemicolectomy with complete mesocolic excision

**DOI:** 10.1007/s00384-021-03891-0

**Published:** 2021-03-01

**Authors:** Gabriele Anania, Alberto Arezzo, Richard Justin Davies, Francesco Marchetti, Shu Zhang, Salomone Di Saverio, Roberto Cirocchi, Annibale Donini

**Affiliations:** 1 Department of Medical Sciences, University of Ferrara, Via Fossato di Mortara 70, 44121 Ferrara, Italy; 2 Department of Surgical Sciences, University of Torino, Torino, Italy; 3 Cambridge Colorectal Unit, Addenbrooke’s Hospital, Cambridge University Hospitals NHS Foundation Trust, Cambridge, UK; 4 Department of Surgery, Fudan University Shanghai Cancer Center, Shanghai, People’s Republic of China; 5 Department of General Surgery, University of Insubria, Varese, Italy; 6 Department of General Surgery, University of Perugia, Perugia, PG Italy

**Keywords:** Right hemicolectomy, CME colectomy, Laparoscopic surgery, Open surgery

## Abstract

**Purpose:**

The aim of this study was to compare the outcomes of right hemicolectomy with CME performed with laparoscopic and open surgery.

**Methods:**

PubMed, Scopus, Web of Science, China National Knowledge Infrastructure, Wanfang Data, Google Scholar and the ClinicalTrials.gov register were searched. Primary outcome was the overall number of harvested lymph nodes. Secondary outcomes were short and long-term course variables. A meta-analysis was performed to calculate risk ratios.

**Results:**

Twenty-one studies were identified with 5038 patients enrolled. The difference in number of harvested lymph nodes was not statistically significant (MD 0.68, − 0.41–1.76, *P* = 0.22). The only RCT shows a significant advantage in favour of laparoscopy (MD 3.30, 95% CI − 0.20–6.40, *P* = 0.04). The analysis of CCTs showed an advantage in favour of the laparoscopic group, but the result was not statically significantly (MD − 0.55, 95% CI − 0.57–1.67, *P* = 0.33). The overall incidence of local recurrence was not different between the groups, while systemic recurrence at 5 years was lower in laparoscopic group. Laparoscopy showed better short-term outcomes including overall complications, lower estimated blood loss, lower wound infections and shorter hospital stay, despite a longer operative time. The rate of anastomotic and chyle leak was similar in the two groups.

**Conclusions:**

Despite the several limitations of this study, we found that the median number of lymph node harvested in the laparoscopic group is not different compared to open surgery. Laparoscopy was associated with a lower incidence of systemic recurrence.

**Supplementary Information:**

The online version contains supplementary material available at 10.1007/s00384-021-03891-0.

## Introduction

Colorectal cancer (CRC) is one of the most common health-threatening diseases around the globe. It is the third most frequent cancer worldwide (1.85 million new cases/year, representing 10.2% of total malignancies) and, according to predictions, is expected to increase in incidence by a further 20% before 2030 [1]. The number of annual worldwide deaths from CRC was approximately 880,000 in 2018, with an increasing trend year on year [2].

The prognosis of this disease is strongly related to the stage at the time of diagnosis, with a 5-year survival rate of around 90% when the cancer is diagnosed at an early stage, compared with 13% when the presentation is delayed and metastatic disease is present [2]. In particular, lymph node involvement determines important variations in outcome, with overall 5-year CRC survival at 59% [3]. Patients with stage II and stage III CRC treated with potentially curative surgery will still sadly die of the disease in up to 30% of cases at 5 years. At least part of this may be determined by understaging of the disease due to an insufficient lymph node yield [4], as this factor is important in determining subsequent oncologic adjuvant treatment [5].

Surgery is the mainstay of potentially curative treatment, also playing a central role in staging. Standard segmental colectomy with D2 lymphadenectomy is based on the oncological principle that local control of disease determines survival, with lymphadenectomy mainly meant for prognosis rather than cure [6]. In fact, the indication for adjuvant chemotherapy is based on several factors, including nodal status, and may provide a reduction in mortality by up to 30%. According to guidelines [7], a minimum of 12 lymph nodes should be analysed for an accurate staging of the disease, whilst an understaging may result in patients not receiving adjuvant therapy.

It is in this context that in 2009 Hohenberger proposed to extend the lymphadenectomy, describing complete mesocolic excision (CME) for the treatment of cancer of the caecum and ascending colon [8]. In subsequent years, the technique spread, and it was later adapted to laparoscopy which had become the accepted standard of care in the surgical treatment of colon cancer. The aim of this systematic review and meta-analysis is to compare short-term and long-term outcomes of individuals with right colon cancer undergoing treatment by open or laparoscopic right hemicolectomy with CME.

## Materials and methods

We performed a systematic review of the literature, which was searched up to 20 March 2020, according to the Preferred Reporting Items for Systematic Reviews and Meta-Analyses (PRISMA) guidelines [9], including the following databases: Medline/PubMed, Scopus, Web of Science (WOS), China National Knowledge Infrastructure (CNKI, 中国知网), Wanfang Data (万方)) and Google Scholar.

The following search statement was used in Medline/PubMed:

- (“laparoscopy”[MeSH Terms] OR “laparoscopy”[All Fields] OR “laparoscopic”[All Fields]) AND (“Vet Surg”[Journal] OR “vs”[All Fields]) AND open[All Fields] AND right[All Fields] AND CME[All Fields] (“laparoscopy”[MeSH Terms] OR “laparoscopy”[All Fields] OR “laparoscopic”[All Fields]) AND (“Vet Surg”[Journal] OR “vs”[All Fields]) AND open[All Fields] AND complete[All Fields] AND mesocolic[All Fields] AND excision[All Fields]
- (“laparoscopy”[MeSH Terms] OR “laparoscopy”[All Fields] OR “laparoscopic”[All Fields]) AND CME[All Fields] AND open[All Fields] AND right[All Fields] AND (“colectomy”[MeSH Terms] OR “colectomy”[All Fields])
- (“laparoscopy”[MeSH Terms] OR “laparoscopy”[All Fields] OR “laparoscopic”[All Fields]) AND open[All Fields] AND central[All Fields] AND (“blood vessels”[MeSH Terms] OR (“blood”[All Fields] AND “vessels”[All Fields]) OR “blood vessels”[All Fields] OR “vascular”[All Fields]) AND (“ligation”[MeSH Terms] OR “ligation”[All Fields]) AND right[All Fields] AND (“colectomy”[MeSH Terms] OR “colectomy”[All Fields])

In the other databases (WOS, Scopus, CNKI and Wanfang Data), the search was performed by entering the association of the following keywords:

- laparoscopic AND open AND right AND colectomy
- laparoscopic AND CME AND open AND right AND colectomy
- laparoscopic AND complete mesocolic excision AND open AND right AND colectomy

A further search was performed through the reference lists of the selected articles and relevant grey literature on Google Scholar. Finally, ClinicalTrials.gov was searched to evaluate any ongoing registered clinical trials.

### Eligibility criteria

Studies that compared participants enrolled for either laparoscopic or open right hemicolectomy with CME were eligible for inclusion. Randomized controlled trials (RCTs) and clinical control studies (CCTs) (prospective and retrospective cohort studies) were included. Case report studies were excluded.

### Study selection and data extraction

Two reviewers (RC and SZ) performed the search independently. A third author (AA) arbitrated any disagreements on inclusion or exclusion of studies. The reference lists of the included studies were searched manually. Only the data for patients who had undergone open or laparoscopic CME were included.

### Outcomes of interest

Primary outcome was the overall number of harvested lymph nodes. Secondary outcomes were local and systemic recurrence at 3 and 5 years, operative time, post-operative mortality at 30 days, overall post-operative complications at 30 days, estimated blood loss, surgical intraoperative complications (vascular injuries, iatrogenic small bowel perforation), anastomotic leak rate, chyle leak rate, post-operative hospital stay, post-operative ileus, wound infections and pulmonary infections.

### Quality assessment

All studies fulfilling the selection criteria for this systematic review and meta-analysis were assessed for methodological quality and risk of bias by two authors (RC and GA). The individual scores of quality assessment items per study were assessed using the Cochrane risk tool for Randomised Control Trials [10, 11] and the methodological index for non-randomised studies (MINORS) [12].

### Statistical analyses

All analyses were performed according to original treatment allocation (intention-to-treat analysis). The categorical variables were described as absolute/relative frequencies and the continuous ones as median and interquartile range (IQR). Data were analysed for risk ratios (RR) in the case of dichotomous variables, and weighted mean differences (WMD) for continuous variables. The randomised Mantel-Haenszel method was used for the meta-analysis. All results were displayed in Forest plots. The *I*^2^ and the Cochrane’s Q were reported as statistical measures of heterogeneity. For outcomes with more than ten studies, funnel plots are shown. Other statistical measures of bias are not reported given the high study heterogeneity. The data analysis was performed using the meta-analysis software Review Manager (RevMan) v 5.3.5 (Copenhagen: The Nordic Cochrane Centre, The Cochrane Collaboration, 2018).

In this meta-analysis, a subgroup analysis according to the types of study design was performed. We also identified the studies which had both the largest variance (wide intervals) and the extreme outlier weight in each clinical outcome group.

## Results

### Study selection

**Fig. 1:**
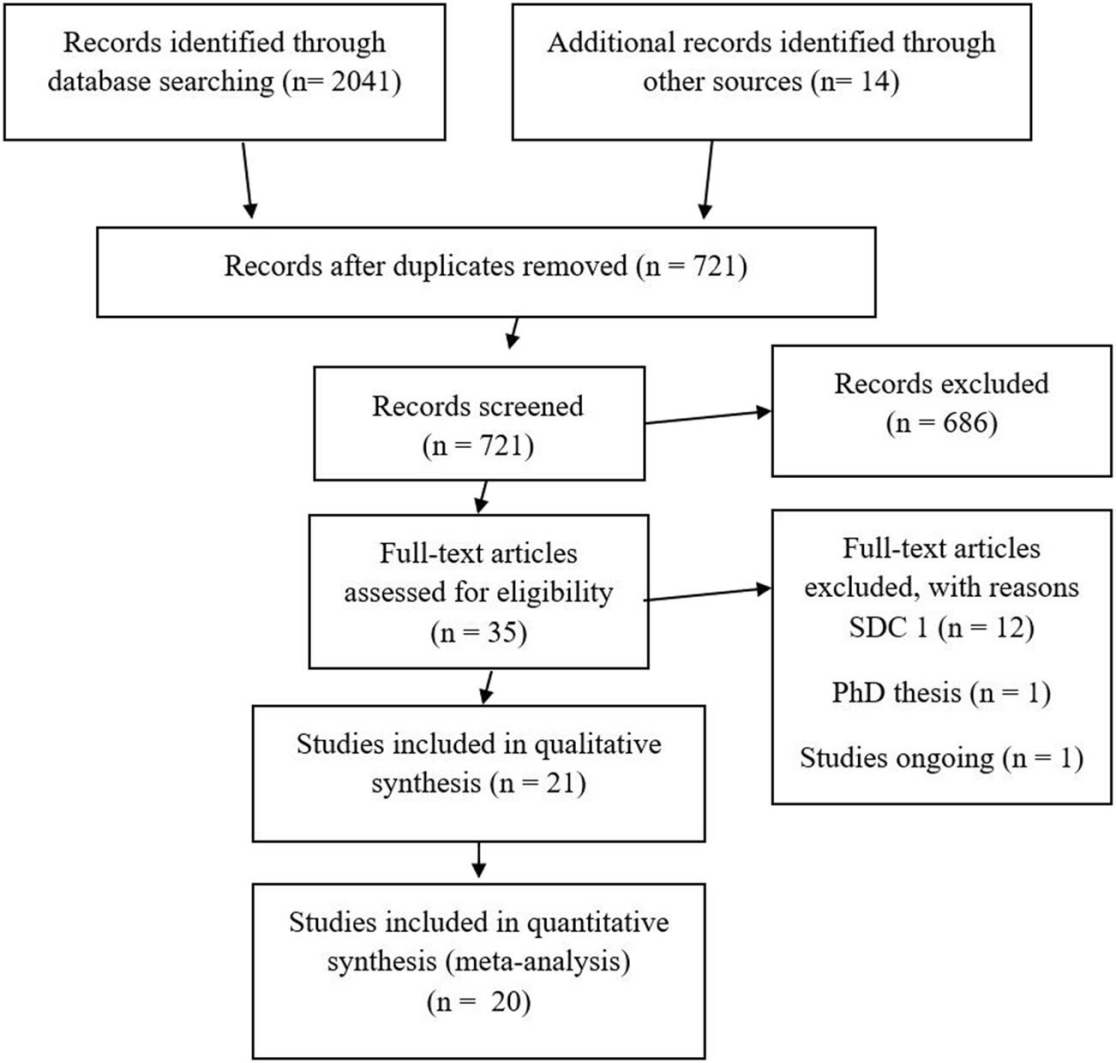
Prisma flow chart of literature search

The PRISMA flow diagram is presented in Fig. 1. The initial search produced 2055 studies. After removal of duplicates, 721 citations remained. After screening of titles and abstracts, 36 studies were analysed in full text, with 12 studies excluded as reported in SDC1, leaving 23 studies matching the inclusion criteria for meta-analysis [13–47], one PhD thesis by El Nakeeb (not published) [13]. Of these, two references [15, 16] report the same data as well as the PhD thesis [13]. In addition, we found one ongoing study (registered in ClinicalTrials.gov as NCT03826446) [48].

Altogether, the 21 studies included provided data on 5038 patients (Table 1). The included studies were published between 2010 and 2020, with participants enrolled between 2000 [19, 34] and 2019 [13, 15] (Table 1).

**Table 1:** Inclusion criteria

Author–year of publication		Nation	Type of study	No. of patients included	Time of enrolment	Type of access	No. of patients for access (laparosopic/open)	
Wang 2020		China	R	280	2010–2015	LA (M)-OA	160	120
Menoufia University	Elbalshy 2019	Egypt	RCT	60	2016–2019	LA (M)-OA	30	30
	El Fol 2019							
Jin 2019		China	R	153	2011–2015	LA (M)-OA	71	82
Pelz 2018		Germany	R	279	2009–2016	LA (M)-OA	24	255
Shin 2018		South Korea	P	2249	2000–2013	LA (M)-OA	1010	1239
Yu 2018		China	R	218	2010–2014	LA (M)-OA	102	116
Li 2018		China	R	88	2012–2015	LA (M)-OA	40	48
Aiypov 2018		Russia	R	59	NR	LA (M)-OA	11	48
Rasulov 2017		Russia	P	39	2015–2016	LA (M)-OA	22	17
Chen 2017		China	R	82	2011–2012	LA (M)-OA	27	55
Mondal 2017		Bangladesh	P	24	2015–2017	LA (M)-OA	14	10
Huang 2015		China	R	102	2012–2013	LA (M)-OA	53	49
Yin 2015		China	R	267	2010–2014	LA (M)-OA	75	192
Liu 2015		China	R	84	2011–2012	LA (M)-OA	44	40
Gao 2015		China	R	55	2010–2014	LA (M)-OA	18	37
Zhao G 2014		China	R	46	2010–2013	LA (M)-OA	24	22
Cong 2014		China	R	178	2008–2011	LA (M)-OA	96	82
Bae 2014		South Korea	R	170	2006–2008	LA (M)-OA	85	85
Zhao L 2014		China	R	220	2006–2009	LA (M)-OA	119	101
Han 2014		China	R	324	2003–2010	LA (M)-OA	177	147
Guan 2010		China	R	61	2006–2010	LA (M)-OA	29	32

Type of study: *RCT*, randomized control study; *R*, observational retrospective; *P*, observational prospective

Type of approach: *LA*, laparoscopic assisted; *OA*, open access; *M*: multiport

### Study characteristics

There were one RCT, performed at Menoufia University (Egypt) [15, 16] and 20 CCTs [14, 17–35]. Sixteen studies were performed in Asia (4601 patients, 91.3%), including 13 from China (2158 patients), 2 from South Korea (2419 patients) and 1 from Bangladesh (24 patients). Three studies were performed in Europe (377 patients, 7.5%), including 2 in Russia (102 patients) and 1 in Germany (279 patients). One study was performed in Egypt (Africa) (60 patients, 1.2%). Pooled trials were comparable for age, gender, BMI (body mass index), ASA (American Society of Anesthesiology) and TNM stage (SDC 2). Inclusion and exclusion criteria were mostly well reported but varied considerably between studies.

Among the examined studies, patients with stage IV disease were excluded, except for one [18]. The other studies include stages I, II and III, but two [14, 25] only included stage III cancers and two [22, 26] only included stages II and III; in addition, five studies [15, 28–30, 32] do not indicate the TNM stage.

Concerning tumour localisation, all of the studies considered cancers of cecum, ascending colon and hepatic flexure, while only five studies [17, 20, 22, 31, 32] also included proximal transverse colonic neoplasms.

For what it concerns conversion from laparoscopic to open resection, most of the studies excluded the patients which required this procedure for any reason. From those other studies that considered in their data also converted surgeries, we excluded the converted procedures in our analysis.

### Quality assessment

We assessed the risk of bias for each trial and summarised them using the criteria and the ‘Risk of bias’ Table (SDC 3). No data were reported on random sequence generation or allocation concealment in the RCT, while blinding of participants and personnel was not reported. A ‘low risk of bias’ was reported in the analysis of the attrition bias and an ‘unclear risk of bias’ for selective reporting. Risks of bias assessed with the MINORS scale quality assessment for non-RCTs is reported in SDC 4. The mean score was 16 (moderate risk).

### Primary outcome

The overall number of harvested lymph nodes was reported in 3876 patients (19 studies) [14, 15, 17–21, 24–35]. The number of nodes was higher in the laparoscopic group, but the difference was not statistically significant (MD 0.68, − 0.41–1.76, *P* = 0.22, *I*^2^ = 90%).

**Fig. 2:**
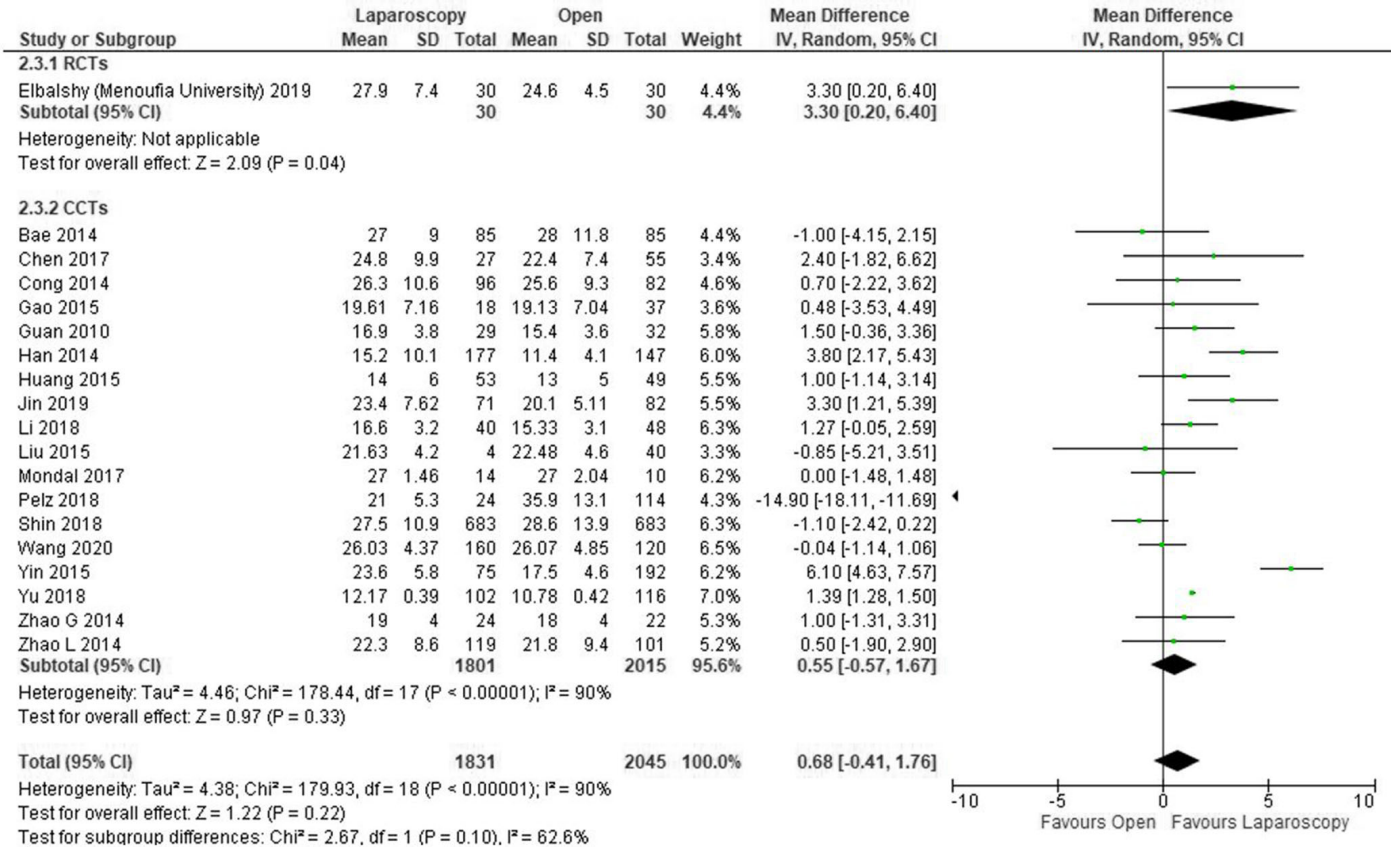
Forest plot of comparison: Laparoscopic versus open CME right hemicolectomy. Overall number of harvested lymph nodes

The analysis of the RCT performed from Menoufia University (Egypt) shows a significant advantage in favour of laparoscopy (MD 3.30, 95% CI − 0.20–6.40, *P* = 0.04). However, the analysis of CCTs showed an advantage in favour of the laparoscopic group, but not statically significant (MD 0.55, 95% CI − 0.57–1.67, *P* = 0.33, *I*^2^ = 90%) (Fig. 2).

### Secondary outcomes

Overall recurrence at 3 years was reported in 3 studies [14, 16, 24] (*n* = 414). The incidence of overall recurrence was significantly lower in the laparoscopic group (RR 0.55, 95% CI 0.34 to 0.91, *P* = 0.02, *I*^2^ = 0%) (SDC 5).

Overall recurrence at 5 years was reported in 3 studies [19, 31, 33] (*n* = 1860). The incidence of overall recurrence was significantly lower in the laparoscopic group (RR 0.61, 95% CI 0.48 to 0.77, *P* = < 0.0001, *I*^2^ = 0%) (SDC 6).

Local recurrence at 3 years was reported in 3 studies [14, 16, 24] (*n* = 414). The overall incidence of local recurrence was significantly lower in the laparoscopic group (RR 0.60, 95% CI 0.38 to 0.95, *P* = 0.03, *I*^2^ = 0%) (SDC 7).

Local recurrence at 5 years was reported in 4 CCTs [19, 29, 31, 33] (*n* = 1944). The overall incidence of local recurrence was not different between the groups (RR − 0.55, 95% CI 0.20 to 1.54, *P* = 0.26, *I*^2^ = 54%) (SDC 8).

Systemic recurrence at 3 years was reported in 2 CCTs [14, 24] (*n* = 354). The overall incidence of systemic recurrence was not different between the groups (RR 1.13, 95% CI 0.31–4.11, *P* = 0.85, *I*^2^ = 0%) (SDC 9).

Systemic recurrence at 5 years was reported in 3 CCTs [19, 31, 33] (*n* = 1860). The overall incidence of systemic recurrence was significantly lower in laparoscopic group (RR 0.53, 95% CI 0.39–0.72, *P* = 0.001, *I*^2^ = 28%) (SDC 10).

**Fig. 3:**
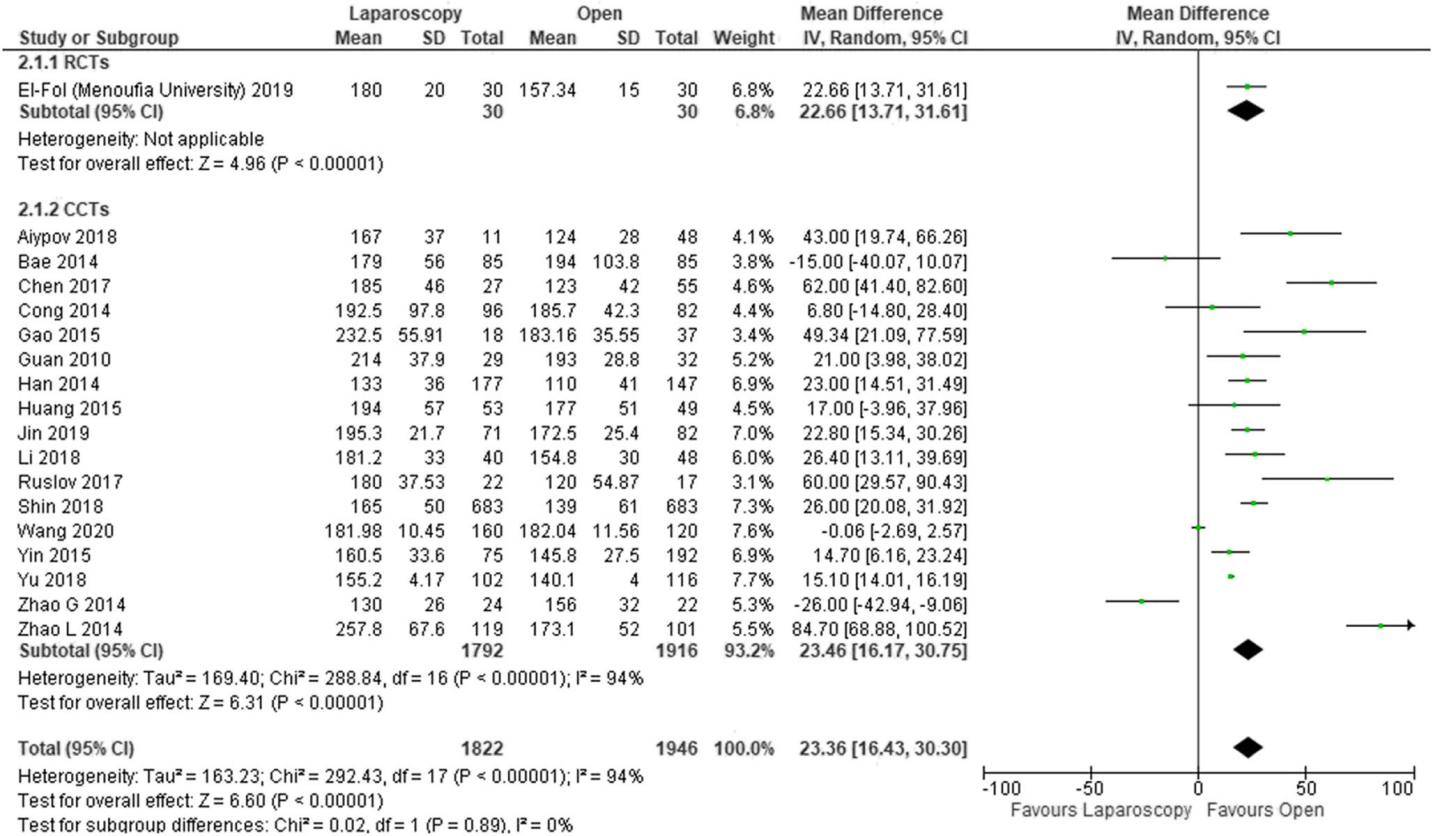
Forest plot of comparison: Laparoscopic versus open CME right hemicolectomy. Operative time

Twenty studies [14, 16, 17, 19–24, 26–28, 30–35] reported operative time (3768 patients). This was significantly longer in the laparoscopic group compared to open surgery (MD − 23.26, 95% CI − 16.17 to − 30.75, *P* < 0.00001; *I*^2^ = 94%) (Fig. 3).

Nine studies [16, 19, 21, 22, 24, 27, 31–33] reported post-operative mortality at 30 days (*n* = 2471). No statistically significant difference was observed between the two groups (RR 0.53, 95% CI 0.13–2.11, *P* = 0.37, *I*^2^ = 0%) (SDC 11).

**Fig. 4:**
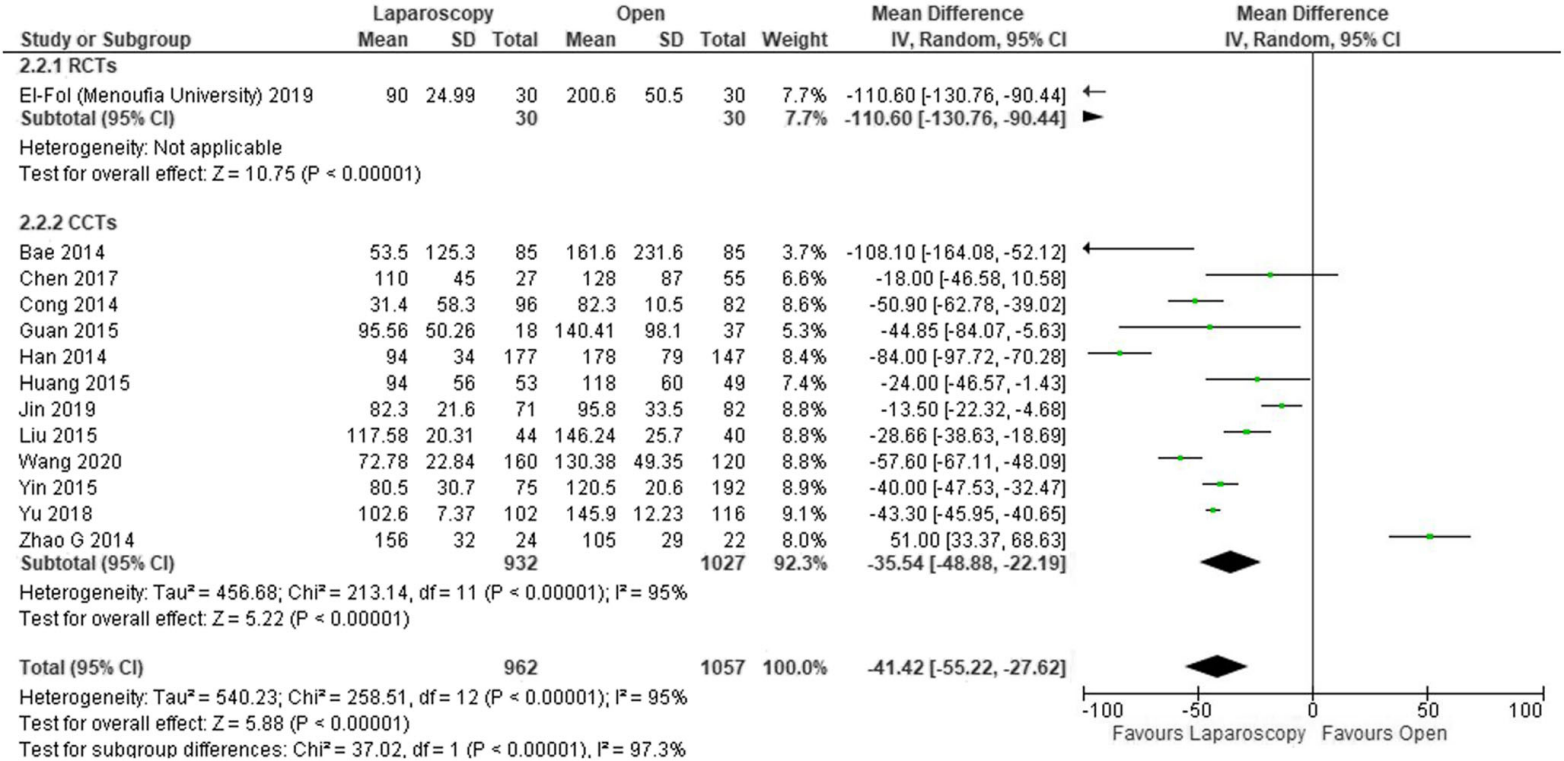
Forest plot of comparison: Laparoscopic versus open CME right hemicolectomy. Intraoperative blood loss

Intraoperative blood loss was reported in 13 studies [14, 16, 17, 20, 24, 26–29, 31, 33–35] (2139 patients). The estimated blood loss was statistically significantly lower in the laparoscopic group compared with the open group (MD − 41.42, 95% CI − 52.22 to − 27.62, *I*^2^ = 95%) (Fig. 4).

Four studies [23, 25, 32, 33] reported on surgical intraoperative complications (vascular injuries, iatrogenic small bowel perforation) (607 patients). These were lower in the open (0.36%, 1/275) than in the laparoscopic group (1.2%, 4/332), but the result was not statistically significant (RR 1.72, 95% CI 0.38 to 7.85; *I*^2^ = 0%) (SDC 12).

Twelve studies [16, 17, 19–21, 24, 27, 28, 30–33] reported post-operative complications at 30 days (2991 patients). These were significantly lower in the laparoscopic group (RR 0.83, 95% CI 0.71–0.97, *P* = 0.02, *I*^2^ = 0%) (SDC 13).

**Fig. 5:**
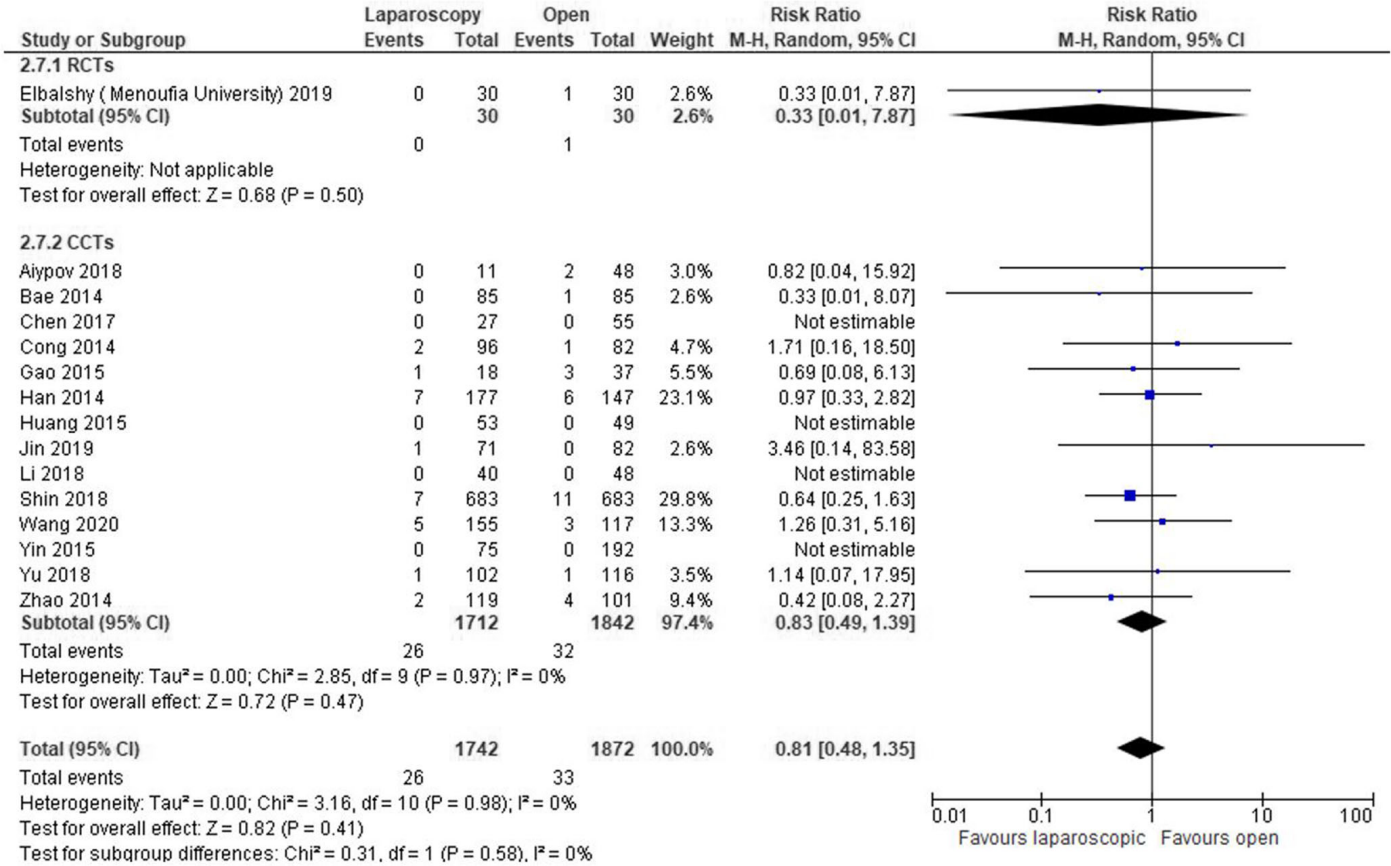
Forest plot of comparison: Laparoscopic versus open CME right hemicolectomy. Anastomotic leak

Anastomotic leak (AL) was reported in 15 studies [14, 15, 17, 19–22, 24, 26–28, 30–33] (3614 patients). No statistically significant difference was observed between the two groups (RR 0.81, 95% CI 0.48–1.35, *P* = 0.47, *I*^2^ = 0%) (Fig. 5).

**Fig. 6:**
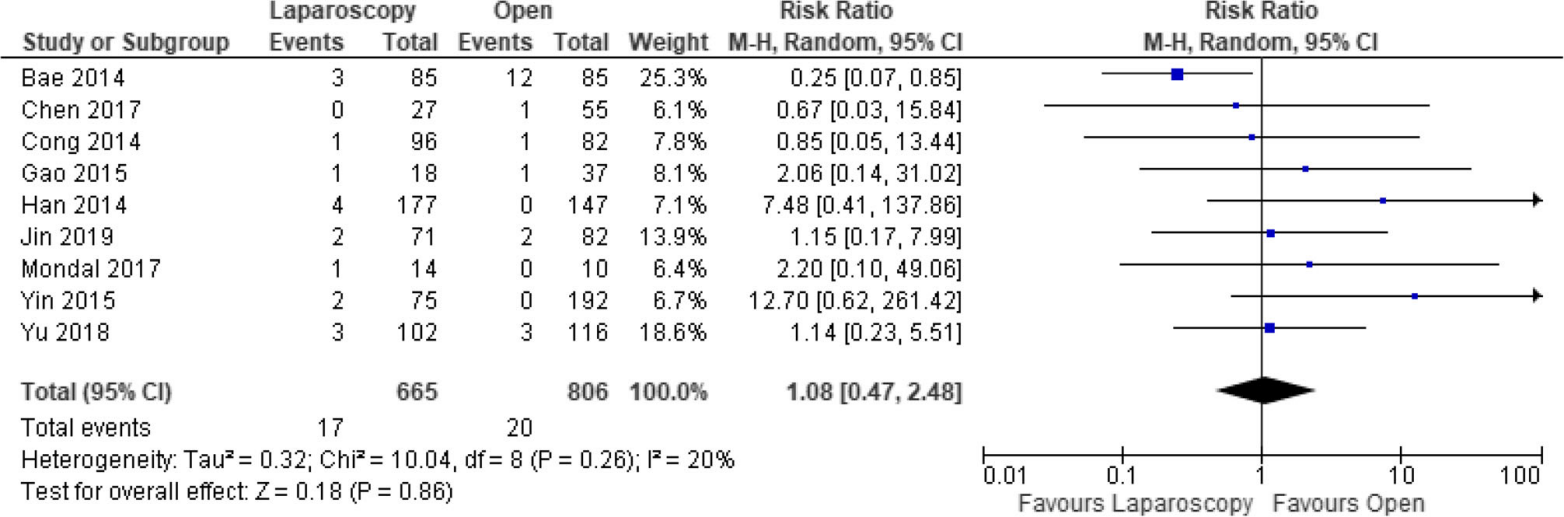
Forest plot of comparison: Laparoscopic versus open CME right hemicolectomy. Chylous leak

Nine studies [17, 20, 24–26, 28, 30, 31, 33], all CCTs, reported on rates of chyle leak (*n* = 1293). These did not differ between groups (RR 1.08, 95% CI 0.47–2.48, *P* = 0.86, *I*^2^ = 20%) (Fig. 6).

Six studies [17, 20, 24, 28, 30, 34], all CCTs, reported post-operative hospital stay (*n* = 821). This was significantly lower in the laparoscopic group compared to the open group (MD − 2.68, 95% CI − 4.10–1.26, *P* = 0.0002, *I*^2^ = 92%) (SDC 14).

Ten studies [16, 17, 19–21, 24, 26, 28, 31, 33] reported on post-operative ileus (*n* = 2906). No statistically significant difference was observed between the two groups (RR 1.05, 95% CI 0.76–1.44, *P* = 0.79, *I*^2^ = 0%) (SDC 15).

Eigth studies [16, 17, 21, 24, 26, 28, 31, 33] reported on post-operative wound infections (*n* = 1322). These were significantly fewer in the laparoscopic group compared to the open group (RR 0.41, 95% CI 0.22–0.79, *P* = 0.007, *I*^2^ = 0%) (SDC 16).

Four studies [14, 21, 24, 33] reported pulmonary infections (*n* = 774). No statistically significant difference was observed between the two groups (RR 0.50, 95% CI 0.50–1.70, *P* = 0.27, *I*^2^ = 46%) (SDC 17).

## Discussion

The CME technique as part of a right hemicolectomy is technically difficult because of the large variability in the right colon vasculature, which may be the cause of challenging intraoperative bleeding complications. Moreover, CME should be performed by surgeons with a significant experience in colorectal surgery. It is important to recall that the localisation of the disease influences the extent of lymphadenectomy in the peripancreatic area: for experts of the CME technique, cancers located in the hepatic flexure and in proximal transverse colon require extension to the Henle trunk stations.

Several authors have published their data and results in the execution of CME in a laparoscopic setting, which clearly requires further experience in advanced laparoscopic surgery. The choice between laparoscopy or open surgery for CME remains a point of discussion and interest. Moreover, some argue that CME may be an appropriate indication for a robotic approach [49, 50], and further data are required to assess the utility of robotics in this setting.

We compared laparoscopic and open CME in terms of the number of lymph nodes harvested. Moreover, we evaluated the differences in long-term prognosis considering the 3-year and 5-year post-operative overall survival and disease-free survival. Local control of the disease is improved by an increased number of harvested lymph nodes [7]. The samples of patients were comparable for age, gender, BMI, ASA and TNM stage. In addition, despite the majority of the studies indicated the TNM stage, the exact localisation of primary tumour was often not reported. This clearly represents a limit in the literature available regarding this topic. Finally, raw data or hazard ratios of survival curves, both overall and disease-free, would be more useful to understand the true equivalence of laparoscopic and open techniques in terms of oncologic outcome. During the entire analysis, a significant heterogeneity was often observed, so that we preferred to use, as standard, random effects. For these reasons, we would need an Individual Patient Database (IPD) or, at least, the hazard ratios of the published studies.

Laparoscopic CME may allow more lymph nodes to be harvested than in open surgery. Nevertheless, laparoscopy appeared superior to open surgery in terms of overall recurrence at 3 and 5 years, the only measurable parameter related to oncologic adequacy. However, there needs to be caution in interpretation of this result due to the possible influence of selection bias.

Improved staging of the disease does allow patients to be considered for the most appropriate therapeutic approach, in particular the receipt of adjuvant chemotherapy. Han et al. [33] also showed how laparoscopy resulted in an increased harvested number of so-called principal lymph nodes (along the course of the superior mesenteric artery), this, being more radical, and possibly aided by the improved magnification of the surgical field offered by laparoscopy.

Besides extended lymphadenectomy, CME requires the mesocolic fascia to be excised intact, in order to guarantee advantages in term of oncological radicality and survival [8, 51–53]. In our systematic review and meta-analysis, we did not report data about the quality of the surgical specimen, since only five studies [20, 23, 26, 28, 30], among those included, explicitly reported this feature. In fact, most of the authors, although describing in detail the technique used and highlighting the importance of the integrity of the mesocolon, focused their attention on the number of harvested lymph nodes in order to evaluate the local control of the disease. This bias could derive from low initial experience of pathologists in the examination of the mesocolic fascia, in a similar way that happened at the beginning of the application of total mesorectal excision (TME) for rectal cancer. However, it remains technically imperative to maintain the integrity of the mesocolic fascia during CME in order to maximise appropriate oncological radicality.

It is important to remember that laparoscopic approach may fail and require conversion to open surgery because of various reasons: the main factor that implicates conversion is a vascular injury that leads to uncontrollable bleeding or that cannot be detected in its source; other conditions that could require the change of procedure could be the excess of adhesions or an organ injury hard to repair laparoscopically. In case of conversion to the open approach, there are consequences for the patient that must be considered and discussed before choosing this method: in particular, the outcome of the patient worsens in term of longer and complicated post-operative stay, the possible need for post-operative ICU and longer operative time. These complications are a direct consequence of the conversion and are not a consequence of the open technique, that is why it is very important to select the patients that will undergo laparoscopic surgery [54, 55].

The presented data show laparoscopy to be at least non inferior to open surgery when performing CME for cancer of the right colon, with other benefits of a minimally invasive approach also confirmed. Moreover, the laparoscopy group resulted in a lower 5-year systemic recurrence rate, even if this outcome was reported by only a few studies: the results might be considered promising but will need further confirmation by the enlargement of the cohort of studies. Laparoscopy offered better short-term outcomes including overall complications, lower estimated blood loss and lower wound infection rates, altogether translating into a shorter hospital stay. This appears at the expense of a longer operative time, likely due to the higher technical difficulty associated with the surgery, in particular when a laparoscopic extended right hemicolectomy is performed for tumours located at the hepatic flexure or proximal transverse colon. These endpoints are similar to other meta-analyses [37, 38] regarding this topic, and a possible explanation is that the laparoscopic technique is harder to perform and requires a longer learning curve. No difference was observed in terms of post-operative mortality, anastomotic leak, chylous leak or pulmonary infection, which is reassuring.

The current study has several limitations. Although this systematic review included the highest number of studies reported in literature, the major limitation is represented by the geographical distribution of the studies. In fact, all but one studies which was performed in Germany [18] were published by non-western groups: 16 studies were from Asia (13 from China, 2 from South Korea and 1 from Bangladesh), 1 from Africa (Egypt) and 2 East Europe (Russia). The importance of this limitation is related to the possibility of differences not only in surgical standards but also in standards of adjuvant therapy and health care systems, difficult to assess. A second limitation is that among these studies only one is an RCT. Therefore, the meta-analysis is based on non randomised controlled trials. It would have been interesting to perform a subgroup analysis for TNM/UICC stage associated with the rate of laparoscopic and open surgery, in order to assess possible advantages of laparoscopy in terms of recurrence for a specific tumour stage which requires adjuvant chemotherapy. A third limitation is the impossibility to assess and meta-analyse the data regarding the number of cases in which the minimum of 12 lymph nodes were harvested, corresponding to current guidelines [7]. In order to achieve both these analyses, probably an Individual Participant Database analysis should be planned. Furthermore, it is likely that the control group (open surgery) is often not treated by a well-trained surgeon, being the operation time quite long (median 173 min). Finally, despite Hohenberger [8] recommended a minimum of 25 lymph nodes harvested, the overall median in the open group is only 21.8, with 5 papers reporting even a median of 15 or less.

Another developing field in the group of minimally invasive techniques is robotic surgery: some studies [56, 57] analysed the feasibility, safety and effectiveness of robotic CME, which could be considered as another possible technique to assist CME and having potential for improving the outcomes and quality of life of patients, such as many studies also suggest for laparoscopic procedures as compared to traditional open surgery [58, 59]. However, data are still missing, and the number of studies needs to be increased in order to evaluate this alternative minimally invasive approach.

In conclusion, the present systematic review and meta-analysis fails to show that a laparoscopic approach appears non-inferior to open surgery in terms of lymph nodes harvested but showed a benefit in terms of incidence of systemic recurrence, when performing right hemicolectomy with CME for colon cancer, while maintaining the usual benefits of a minimally invasive surgical approach.

Nevertheless, further prospective and randomised studies are awaited to increase the quantity of data and quality of the evidence.

## Supplementary Information

ESM 1 (DOCX 15 kb)

ESM 2 (DOCX 53 kb)

ESM 3 (DOCX 24 kb)

ESM 4 (DOCX 27 kb)

ESM 5 (DOCX 19 kb)

ESM 6 (DOCX 27 kb)

ESM 7 (DOCX 20 kb)

ESM 8 (DOCX 19 kb)

ESM 9 (DOCX 20 kb)

ESM 10 (DOCX 29 kb)

ESM 11 (DOCX 20 kb)

ESM 12 (DOCX 31 kb)

ESM 13 (DOCX 22 kb)

ESM 14 (DOCX 31 kb)

ESM 15 (DOCX 30 kb)

ESM 16 (DOCX 20 kb)

## Data Availability

All data and materials as well as software application or custom code support the published claims and comply with field standards.

## References

[1] Mattiuzzi C, Sanchis-Gomar F, Lippi G (2019). Concise update on colorectal cancer epidemiology. Ann Transl Med.

[2] International Agency for Research on Cancer, Global Cancers Observatory: Fact sheets cancers. Available at http://gco.iarc.fr/today/fact-sheets-cancers. Accessed 24 march 2020

[3] Lu JY, Xu L, Xue HD, Zhou WX, Xu T, Qiu HZ, Wu B, Lin GL, Xiao Y (2016). The radical extent of lymphadenectomy - D2 dissection versus complete mesocolic excision of LAparoscopic right colectomy for right-sided colon cancer (RELARC) trial: study protocol for a randomized controlled trial. Trials.

[4] O’Connell JB, Maggard MA, Ko CY (2004). Colon cancer survival rates with the new American Joint Committee on Cancer sixth edition staging. J Natl Cancer Inst.

[5] American Cancer Society: Treating Colorectal Cancer. Available at https://www.cancer.org/cancer/colon-rectal-cancer/treating.html. Accessed 24 march 2020

[6] Nelson H, Petrelli N, Carlin A, Couture J, Fleshman J, Guillem J, Miedema B, Ota D, Sargent D (2001). Guidelines 2000 for colon and rectal cancer surgery. J Natl Cancer Inst.

[7] Vogel JD, Eskicioglu C, Weiser MR, Feingold DL, Steele SR (2017). The American Society of Colon and Rectal Surgeons Clinical Practice Guidelines for the treatment of colon cancer. Dis Colon Rectum.

[8] Hohenberger W, Weber K, Matzel K, Papadopoulos T, Merkel S (2009). Standardized surgery for colonic cancer: complete mesocolic excision and central ligation--technical notes and outcome. Color Dis.

[9] Moher D, Liberati A, Tetzlaff J, Altman DG (2009). Preferred reporting items for systematic reviews and meta-analyses: the PRISMA statement. J Clin Epidemiol.

[10] Higgins JP, Altman DG, Gotzsche PC, Juni P, Moher D, Oxman AD, Savovic J, Schulz KF, Weeks L, Sterne JA (2011). The Cochrane Collaboration’s tool for assessing risk of bias in randomised trials. Br Med J.

[11] Higgins JPT, Sterne JAC (2017) Chapter 8: Assessing risk of bias in included studies. Cochrane handbook for systematic reviews of interventions version 520 (updated June 2017)

[12] Slim K, Nini E, Forestier D, Kwiatkowski F, Panis Y, Chipponi J (2003). Methodological index for non-randomized studies (minors): development and validation of a new instrument. ANZ J Surg.

[13] El Nakeeb AES, Elhawary AAA. Comparative study between open vs laparoscopic right hemicolectomy with complete mesocolic excision and central lymphadenectomy for right colon cancer Available athttp://main.eulc.edu.eg/eulc_v5/Libraries/Thesis/BrowseThesisPages.aspx?fn=PublicDrawThesis&BibID=12541275. Accessed 24 March 2020

[14] Wang F, Zhang H, Chu H, Zhu K, Zhang L, Meng W, Zhao S, Zhou W, Li X (2020). The clinical outcomes and prognostic analysis of elderly patients with stage III right colon cancer undergo laparoscopic complete mesocolon. Chin J Bases Clin Gen Surg.

[15] Elbalshy MA, El Fol HA, Ammar MS, Hagag MG (2019). Outcomes of laparoscopic assisted versus open complete mesocolic excision for right sided colon cancer. Int Surg J.

[16] El-Fol HA, Ammar MS, Abdelaziz TF, Elbalshy MA, Elabassy MM (2019). Laparoscopic versus open complete mesocolic excision with central vascular ligation in right colon cancer. Int Surg J.

[17] Jin D, Chen G (2019). Complete mesocolic excision for right colon cancer: laparoscopic versus open surgery. Zhejiang Med.

[18] Pelz JOW, Wagner J, Lichthardt S, Baur J, Kastner C, Matthes N, Germer CT, Wiegering A (2018). Laparoscopic right-sided colon resection for colon cancer-has the control group so far been chosen correctly?. World J Surg Oncol.

[19] Shin JK, Kim HC, Lee WY, Yun SH, Cho YB, Huh JW, Park YA, Chun HK (2018). Laparoscopic modified mesocolic excision with central vascular ligation in right-sided colon cancer shows better short- and long-term outcomes compared with the open approach in propensity score analysis. Surg Endosc.

[20] Yu M, Qi Y, Qin S, Mu Y, Luo Y, Qiu Y, Cui R, Zhong M (2018). A retrospective controlled clinical study on laparoscopic versus open complete mesocolic excision for right colon cancer. J Surg Concepts Pract.

[21] Li T, Meng XL, Chen W (2018). Safety and short-term efficacy of a laparoscopic complete mesocolic excision for the surgical treatment of right hemicolon cancer. Clin Surg Res Commun.

[22] Aiypov RT, Safiullin RI, Garipov MR, Feoktistov DV, Tarasov NA, Garipova AA, Garipov RR (2018). Initial results of D3 lymphadenectomy in the surgical treatment of cancer of the right half of the segmented intestine. Creat Surg Oncol.

[23] Rasulov AO, Malikhov AG, Rakhimov OA, Kozlov NA, Malikhova OA (2017). Short-term outcomes of complete mesocolic excision for right colon cancer. Khirurgiia (Mosk).

[24] Chen Z, Sheng Q, Ying X, Chen W (2017). Comparison of laparoscopic versus open complete mesocolic excision in elderly patients with right hemicolon cancer: retrospective analysis of one single cancer. Int J Clin Exp Med.

[25] Mondal SK, Roy S, Uddin MS, Murshec M, Bashar A (2017). Complete mesocolic excision for right sided coloninc carcinoma – our experience in tertiary care hospital. J Surg Sci.

[26] Cong J, Chen C, Feng Y, Ma M, Xia Z, Liu D (2014). Comparison of short-term outcomes between laparoscopic and open complete mesocolic excision/D3 radical operation for stage II/III right hemicolon carcinoma. Chin J Clin Oncol.

[27] Huang Y, Liu JP (2015). Comparison of laparoscopic versus open complete mesocolic excision for right colon cancer. Int J Surg (Chin Med Assoc).

[28] Yin F, Weng Z, Cen H, Tang C (2015). The effect of complete mesocolic excision in the operation of right colon cancer under laparoscope. Chin J Laparosc Surg (Electron Ed).

[29] Liu J (2015). Clinical analysis of laparoscopic versus open complete mesocolic excision for right colon cancer. China J Endosc.

[30] Gao B, Zhang Y, Zhou C, Wang D, Ma J, Tang D, Jiang P, Yuan J, Wang Y, Yang F (2015). Comparison of laparoscopy-assisted complete mesocolic excision and open operation for right-hemi colon cancer: the safety and short-term outcome. Int J Surg (Chin Med Assoc).

[31] Bae SU, Saklani AP, Lim DR, Kim DW, Hur H, Min BS, Baik SH, Lee KY, Kim NK (2014). Laparoscopic-assisted versus open complete mesocolic excision and central vascular ligation for right-sided colon cancer. Ann Surg Oncol.

[32] Zhao LY, Chi P, Ding WX, Huang SR, Zhang SF, Pan K, Hu YF, Liu H, Li GX (2014). Laparoscopic vs open extended right hemicolectomy for colon cancer. World J Gastroenterol.

[33] Han DP, Lu AG, Feng H, Wang PXZ, Cao QF, Zong YP, Feng B, Zheng MH (2014). Long-term outcome of laparoscopic-assisted right-hemicolectomy with D3 lymphadenectomy versus open surgery for colon carcinoma. Surg Today.

[34] Zhao G, Chen L (2014). Clinical efficacy of laparoscopic laparoscopic complete mesocolic excision with a medial-to-lateral approach for right colon cancer. Chin J Dig Surg.

[35] Guan GX, Liu X, Jiang WZ, Chen ZF, Lu HS (2010). Short-term efficacy of laparoscopic-assisted right hemicolectomy with D3 lymph node dissection in colon cancer. Chin J Gastrointest Surg.

[36] Croner R, Hohenberger W, Strey CW (2015). Comparison of open vs. laparoscopic techniques in complete mesocolic excision (CME) during right hemicolectomy. Zentralbl Chir.

[37] Chaouch MA, Dougaz MW, Bouasker I, Jerraya H, Ghariani W, Khalfallah M, Nouira R, Dziri C (2019). Laparoscopic versus open complete mesocolon excision in right colon cancer: a systematic review and meta-analysis. World J Surg.

[38] Alhassan N, Yang M, Wong-Chong N, Liberman AS, Charlebois P, Stein B, Fried GM, Lee L (2019). Comparison between conventional colectomy and complete mesocolic excision for colon cancer: a systematic review and pooled analysis: a review of CME versus conventional colectomies. Surg Endosc.

[39] Lucchi A, Berti P, Gabbianelli C, Alagna V, Guerra M, Corbucci Vitolo G, Vandi F, Garulli G (2018). Totally laparoscopic right colectomy with complete mesocolon excision. Eur J Surg Oncol.

[40] Negoi I, Hostiuc S, Negoi RI, Beuran M (2017). Laparoscopic vs open complete mesocolic excision with central vascular ligation for colon cancer: a systematic review and meta-analysis. World J Gastrointest Oncol.

[41] Sheng QS, Pan Z, Chai J, Cheng XB, Liu FL, Wang JH, Chen WB, Lin JJ (2017). Complete mesocolic excision in right hemicolectomy: comparison between hand-assisted laparoscopic and open approaches. Ann Surg Treat Res.

[42] Yang X, Wu Q, Jin C, He W, Wang M, Yang T, Wei M, Deng X, Meng W, Wang Z (2017). A novel hand-assisted laparoscopic versus conventional laparoscopic right hemicolectomy for right colon cancer: study protocol for a randomized controlled trial. Trials.

[43] Kim IY, Kim BR, Kim YW (2016). The short-term and oncologic outcomes of laparoscopic versus open surgery for T4 colon cancer. Surg Endosc.

[44] Athanasiou CD, Markides GA, Kotb A, Jia X, Gonsalves S, Miskovic D (2016). Open compared with laparoscopic complete mesocolic excision with central lymphadenectomy for colon cancer: a systematic review and meta-analysis. Color Dis.

[45] Arezzo A, Passera R, Ferri V, Gonella F, Cirocchi R, Morino M (2015). Laparoscopic right colectomy reduces short-term mortality and morbidity. Results of a systematic review and meta-analysis. Int J Color Dis.

[46] Munkedal DL, West NP, Iversen LH, Hagemann-Madsen R, Quirke P, Laurberg S (2014). Implementation of complete mesocolic excision at a university hospital in Denmark: an audit of consecutive, prospectively collected colon cancer specimens. Eur J Surg Oncol.

[47] Tagliacozzo S, Tocchi A (1997). Extended mesenteric excision in right hemicolectomy for carcinoma of the colon. Int J Color Dis.

[48] Laparoscopic versus open complete mesocolic excision with central vascular ligation in right colon cancer. Shady, K.M. Available at: https://clinicaltrials.gov/ct2/show/NCT03826446

[49] Ozben V, De Muijnck C, Esen E, Aytac E, Baca B, Karahasanoglu T, Hamzaoglu I (2018). Is robotic complete mesocolic excision feasible for transverse colon cancer?. J Laparoendosc Adv Surg Tech A.

[50] Bae SU, Yang SY, Min BS (2019). Totally robotic modified complete mesocolic excision and central vascular ligation for right-sided colon cancer: technical feasibility and mid-term oncologic outcomes. Int J Color Dis.

[51] Mike M, Kano N (2015). Laparoscopic surgery for colon cancer: a review of the fascial composition of the abdominal cavity. Surg Today.

[52] West NP, Morris EJ, Rotimi O, Cairns A, Finan PJ, Quirke P (2008). Pathology grading of colon cancer surgical resection and its association with survival: a retrospective observational study. Lancet Oncol.

[53] Garcia-Granero A, Pellino G, Giner F, Frasson M, Grifo Albalat I, Sánchez-Guillén L, Valverde-Navarro AA, Garcia-Granero E (2020). A proposal for novel standards of histopathology reporting for D3 lymphadenectomy in right colon cancer: the mesocolic sail and superior right colic vein landmarks. Dis Colon Rectum.

[54] Kang CY, Chaudhry OO, Halabi WJ, Nguyen V, Carmichael JC, Stamos MJ, Mills S (2012). Outcomes of laparoscopic colorectal surgery: data from the nationwide inpatient sample 2009. Am J Surg.

[55] Belizon A, Sardinha CT, Sher ME (2006). Converted laparoscopic colectomy: what are the consequences?. Surg Endosc.

[56] Spinoglio G, Bianchi PP, Marano A, Priora F, Lenti LM, Ravazzoni F, Petz W, Borin S, Ribero D, Formisano G, Bertani E (2018). Robotic versus laparoscopic right colectomy with complete mesocolic excision for the treatment of colon cancer: perioperative outcomes and 5-year survival in a consecutive series of 202 patients. Ann Surg Oncol.

[57] Yozgatli TK, Aytac E, Ozben V, Bayram O, Gurbuz B, Baca B, Balik E, Hamzaoglu I, Karahasanoglu T, Bugra D (2019). Robotic complete mesocolic excision versus conventional laparoscopic hemicolectomy for right-sided colon cancer. J Laparoendosc Adv Surg Tech A.

[58] McCombie AM, Frizelle F, Bagshaw PF, Frampton CM, Hewett PJ, McMurrick PJ, Rieger N, Solomon MJ, Stevenson AR (2018). The ALCCaS trial: a randomized controlled trial comparing quality of life following laparoscopic versus open colectomy for colon cancer. Dis Colon Rectum.

[59] Głowacka-Mrotek I, Tarkowska M, Nowikiewicz T, Jankowski M, Mackiewicz-Milewska M, Hagner W, Zegarski W (2019). Prospective evaluation of the quality of life of patients undergoing surgery for colorectal cancer depending on the surgical technique. Int J Color Dis.

