# Supplementary material for: A global systematic review and meta-analysis on laparoscopic vs open right hemicolectomy with complete mesocolic excision
Source: Int J Colorectal Dis. 2021 Mar 1;36(8):1609–20. doi: 10.1007/s00384-021-03891-0 (PMC8280018; doi:10.1007/s00384-021-03891-0)
Supplement: Supplementary file 1 — (DOCX 15 kb) [file 384_2021_3891_MOESM1_ESM.docx]

| **Author – Year of publication** | **Reason for the exclusion** |
| --- | --- |
| Chaouch 2019 | A systematic review and meta-analysis |
| Alhassan 2019 | A systematic review |
| Lucchi 2018 | The authors do not report the data about the comparation about open vs laparoscopic right CME colectomy |
| Negoi 2017 | A systematic review and meta-analysis |
| Sheng 2017 | The comparation was performed between hand-assisted laparoscopic vs open right colectomy |
| Yang 2017 | A protocol for a RCT comparing hand-assisted laparoscopic vs open right colectomy |
| Kim 2016 | The comparison was performed between laparoscopic vs open colectomy in every colon location |
| Athanasiou 2016 | A systematic review and meta-analysis |
| Arezzo 2015 | A systematic review and meta-analysis |
| Hohenberger 2015 | A comparative case report |
| Munkedal 2014 | The authors reported an evaluation of specimens resected in the mesocolic plane of patients underwent to laparoscopic and open right colectomy |
| Tagliacozzo 1993 | The comparation was performed between central vascular ligation and conventional open right colectomy |

SDC 1: Excluded studies
