# Supplementary material for: A global systematic review and meta-analysis on laparoscopic vs open right hemicolectomy with complete mesocolic excision
Source: Int J Colorectal Dis. 2021 Mar 1;36(8):1609–20. doi: 10.1007/s00384-021-03891-0 (PMC8280018; doi:10.1007/s00384-021-03891-0)
Supplement: Supplementary file 2 — (DOCX 53 kb) [file 384_2021_3891_MOESM2_ESM.docx]

**SDC 2a: Characteristics of the studies**

| **Author – year of publication** | | **Type of access** | **Patients enrolled** | **Age**  **Mean (SD)** | **Sex**  **(M/F)** | **BMI**  **Mean (SD)** | **ASA**  **(I, II, III, IV)** | **TNM stage**  **(I, II, III, IV)** | **Localization (cecum, ascending colon, right flexure, first third of transverse colon)** | **Exclusion criteria for localization (until right colonic flexure or until first third of transverse colon)** |
| --- | --- | --- | --- | --- | --- | --- | --- | --- | --- | --- |
| El Nakeeb 2020 | | Laparoscopy | 30 | 55 | 20/10 | 27 | NR | NR | NR | Only right colonic cancers |
|  |  | Open | 30 | 58.5 | 18/12 | 26.2 | NR | NR | NR |  |
| Wang  2020 | | Laparoscopy | 160 | 71.96±7.18 | 98/62 | 21.26±2.18 | NR | TNM: 0,0,160,0 (100% of stage III) | NR | Until right colonic flexure |
|  |  | Open | 120 | 72.12±6.30 | 67/53 | 21.13±2.06 | NR | TNM: 0,0,120,0 (100% of stage III) | NR |  |
| RCT      Menoufia  University | Elbalshy 2019 | Laparoscopy | 30 | 53.47±11.5 | 19/11 | 27.8 | NR | NR | NR | Until right colonic flexure |
|  |  | Open | 30 | 51.5±9.46 | 17/13 | 25.56 | NR | NR | NR |  |
|  | El Fol 2019 | Laparoscopy | 30 | 58.33±5.88 | 14/16 | NR | NR | TNM:4,12,14,0  (13,3% stage I, 40% stage II, 46,6% stage III) | 10, 14, 6,0 | Until right colonic flexure |
|  |  | Open | 30 | 59.93±5.20 | 14/16 | NR | NR | TNM:4,14,12,0 (13,3% stage I, 46,6% stage II, 40% stage III) | 8, 16, 6,0 |  |
| Jin  2019 | | Laparoscopy | 71 | 70.6±8.35 | 40/31 | NR | NR | TNM: 5,39,27,0 (7% stage I, 54,9% stage II, 38% stage III) | 22,47(ascending+flexure),2 | Until first third of transverse colon |
|  |  | Open | 82 | 71.3±9.26 | 45/37 | NR | NR | TNM: 3,46,33,0 (3,6% stage I, 56,1% stage II, 40,2% stage III) | 28,51(acending+flexure),3 |  |
| Pelz  2018 | | Laparoscopy | 24 | 63.9 ± 13.7 | 12/12 | 25.6 ± 5.5 | 0, 20, 4, 0 | TNM:21 (adenomas),3,0,0,0 (87,4% adenomas, 12,5% stage I) | NR | Until right colonic flexure |
|  |  | Open | 255 | 71.2 ± 12.6 | 118/137 | 26.3 ± 4.6 | 6, 119, 117, 13 | TNM:23 (adenomas),44,77,70,41 (9% adenomas, 17,3% stage I, 30,2% stage II, 27,5% stage III, 16,1% stage IV) | NR |  |
| Shin  2018 | | Laparoscopy | 1010 | 60 ± 11 | 445/565 | 23.0 ± 2.6 | ½: 986 ¾:24 | TNM:290,370,350,0 (28,7% stage I, 36,6% stage II, 34,7% stage III) | NR | Until right colonic flexure |
|  |  | Open | 1239 | 62 ± 13 | 543/696 | 23.2 ± 3.4 | ½: 1207 ¾:32 | TNM:126,603,510,0 (10,2% stage I, 48,7% stage II, 41,1% stage III) | NR |  |
| Yu  2018 | | Laparoscopy | 102 | 61.22±1.13 | 58/44 | NR | NR | TNM:14,51,37,0 (13,7% stage I, 50% stage II, 36,3% stage III) | 18,65,0,19 | Until first third of transverse colon |
|  |  | Open | 116 | 62.58±1.15 | 70/46 | NR | NR | TNM:7,74,35,0 (6% stage I, 63,8% stage II, 30,2% stage III) | 21,62,33 |  |
| Li  2018 | | Laparoscopy | 40 | 59.52 ± 12.42 | 22/18 | NR | NR | TNM: 4, 27, 9, 0 (10% stage I, 67,5% stage II, 22,5% stage III) | NR | Only right colonic cancers |
|  |  | Open | 48 | 60.81 ± 11.63 | 23/25 | NR | NR | TNM:4, 34, 10, 0 (8,3% stage I, 70,8% stage II, 20,8% stage III) | NR |  |
| Aiypov  2018 | | Laparoscopy | 11 | 62 ± 7,2 | 5/6 | NR | NR | TNM: 0,8,3,0 (72,7% stage II, 27,3% stage III) | 4,2,3,2 | Until first third of transverse colon |
|  |  | Open | 48 | 59 ± 8,5 | 25/23 | NR | NR | TNM: 0,31,17,0 (64,6% stage II, 35,4% stage III) | 14,16,11,7 |  |
| Rasulov 2017 | | Laparoscopy | 22 | 59,4 (28-81) | 21/18 | 22,0 (19,9-39,4) | NR | TNM:7, 18, 14, 0 (17,9% stage I, 46,2% stage II, 35,9% stage III) | NR | Only right colonic cancers |
|  |  | Open | 17 |  |  |  | NR |  | NR |  |
| Chen  2017 | | Laparoscopy | 27 | 73.5±5.6 | 18/9 | 23.7±3.2 | 4,10,13,0 | TNM:3,11,13,0 (11,1% stage I, 40,7% stage II, 48,2% stage III) | 0,18,9,0 | Until right colonic flexure |
|  |  | Open | 55 | 75.1±6.4 | 34/21 | 25.1±3.6 | 8,19,28,0 | TNM:7,23,25,0 (12,7% stage I, 41,8% stage II, 45,5% stage III) | 0,35,20,0 |  |
| Mondal  2017 | | Laparoscopy | 14 | NR | NR | NR | NR | TNM: 0,0,14,0 (100% stage III) | 9,5,0,0 | Only cecum and ascending colon |
|  |  | Open | 10 | NR | NR | NR | NR | TNM: 0,0,10,0 (100% stage III) | 7,3,0,0 |  |
| Huang  2015 | | Laparoscopy | 53 | 56 ±7 | 33/20 | NR | NR | TNM:7,26,20,0 (13,2% stage I, 49,1% stage II, 37,7% stage III) | 10,35,8,0 | Until right colonic flexure |
|  |  | Open | 49 | 55 ± 8 | 28/21 | NR | NR | TNM:4,28,17,0 (8,2% stage I, 57,1% stage II, 34,7% stage III) | 8,31,10,0 |  |
| Yin  2015 | | Laparoscopy | 75 | 61.8 | 40/35 | NR | NR | NR | 13,37,25,0 | Until right colonic flexure |
|  |  | Open | 192 | 63.5 | 105/87 | NR | NR | NR | 57,78,57,0 |  |
| Liu  2015 | | Laparoscopy | 44 | 57.2±4.5 | 22/22 | 20.8±1.3 | NR | NR | NR | Only right colonic cancers |
|  |  | Open | 40 | 56.7±5.2 | 21/19 | 21.1±1.7 | NR | NR | NR |  |
| Gao  2015 | | Laparoscopy | 18 | 54.72±12.53 | 9/9 | 20.64±2.13 | NR | NR | NR | Only right colonic cancers |
|  |  | Open | 37 | 61.03±11.24 | 19/18 | 21.58±4.13 | NR | NR | NR |  |
| Zhao G  2014 | | Laparoscopy | 24 | 59±12 | 14/10 | NR | NR | NR | 8,9,7,0 | Until right colonic flexure |
|  |  | Open | 22 | 58±9 | 12/10 | NR | NR | NR | 6,8,8,0 |  |
| Cong  2014 | | Laparoscopy | 96 | 61.5±19.7 | 43/53 | 22.3±3.7 | NR | TNM:0,63,33,0 (65,6% stage II, 34,4% stage III) | NR | Until right colonic flexure |
|  |  | Open | 82 | 62.3±18.4 | 37/45 | 23.5±4.1 | NR | TNM:0,56,26,0 (68,3% stage II, 31,7% stage III) | NR |  |
| Bae  2014 | | Laparoscopy | 85 | 64 | 45/40 | 22.8 | NR | TNM:8,40,37,0 (9,4% stage I, 47,1% stage II, 43,5% stage III) | 15,58,0,12 | Until first third of transverse colon |
|  |  | Open | 85 | 65 | 47/38 | 22.7 | NR | TNM:7,41,37,0 (8,2% stage I, 48,2% stage II, 43,5% stage III) | 13,63,0,9 |  |
| Zhao L  2014 | | Laparoscopy | 119 | 61.3 ± 25.3 | 66/53 | 22.3 ± 3.3 | NR | TNM:6,63,50,0 (5,1% stage I, 52,9% stage II, 42% stage III) | 0,0,89,30 | Until first third of transverse colon |
|  |  | Open | 101 | 64.5 ± 24.6 | 57/44 | 22.6 ± 3.5 | NR | TNM:7,54,40,0 (6,9% stage I, 53,5% stage II, 39,6% stage III) | 0,0,65,36 |  |
| Han  2014 | | Laparoscopy | 177 | 67 ± 12 | 83/94 | NR | NR | TNM: 23,96,58,0 (13% stage I, 54,2% stage II, 32,8% stage III) | NR | Only right colonic cancers |
|  |  | Open | 147 | 65 ± 12 | 80/67 | NR | NR | TNM: 20,68,59,0 (13,6% stage I, 46,3% stage II, 40,1% stage III) | NR |  |
| Guan  2010 | | Laparoscopy | 29 | 60 | 18/11 | NR | NR | TNM: 2,10,17,0 (6,9% stage I, 34,5% stage II, 58,6% stage III) | NR | Only right colonic cancers |
|  |  | Open | 32 | 61 | 20/12 | NR | NR | TNM: 4,7,21,0 (12,5% stage I, 21,9% stage II, 65,6% stage III) | NR |  |

| **Author – year of publication** | | **Correct specimen analysis with evaluation of dissection plan and quality?** |
| --- | --- | --- |
| El Nakeeb 2020 | Laparoscopy | Executed according to principles of CME (clear explanation of procedure), NO explicit report of evaluation of the specimen |
|  | Open |  |
| Wang  2020 | Laparoscopy | Executed according to principles of CME (clear explanation of procedure), NO explicit report of evaluation of the specimen |
|  | Open |  |
| Elbalshy 2019 | Laparoscopy | Executed according to principles of CME (clear explanation of procedure), NO explicit report of evaluation of the specimen |
|  | Open |  |
| El Fol  2019 | Laparoscopy | Executed according to principles of CME (clear explanation of procedure), NO explicit report of evaluation of the specimen |
|  | Open |  |
| Jin  2019 | Laparoscopy | Executed according to principles of CME (clear explanation of procedure), NO explicit report of evaluation of the specimen |
|  | Open |  |
| Pelz  2018 | Laparoscopy | Executed according to principles of CME (clear explanation of procedure), NO explicit report of evaluation of the specimen |
|  | Open |  |
| Shin  2018 | Laparoscopy | Executed according to principles of CME, RADIAL MARGIN WAS NEVER INVOLVED (clear explanation of procedure) |
|  | Open |  |
| Yu  2018 | Laparoscopy | YES, All specimens are checked for mesenteric integrity and cutting edge distance |
|  | Open |  |
| Li  2018 | Laparoscopy | Executed according to principles of CME (clear explanation of procedure), NO explicit report of evaluation of the specimen |
|  | Open |  |
| Aiypov  2018 | Laparoscopy | Executed according to principles of CME (clear explanation of procedure), NO explicit report of evaluation of the specimen |
|  | Open |  |
| Rasulov 2017 | Laparoscopy | YES, CME execution quality was grade I for 2,5% and grade III for 97,5% of patients |
|  | Open |  |
| Chen  2017 | Laparoscopy | Executed according to principles of CME (clear explanation of procedure), NO explicit report of evaluation of the specimen |
|  | Open |  |
| Mondal  2017 | Laparoscopy | Executed according to principles of CME, RADIAL MARGIN WAS NEVER INVOLVED (clear explanation of procedure) |
|  | Open | Executed according to principles of CME, RADIAL MARGIN WAS INVOLVED IN 2 CASES (clear explanation of procedure) |
| Huang  2015 | Laparoscopy | Executed according to principles of CME (clear explanation of procedure), NO explicit report of evaluation of the specimen |
|  | Open |  |
| Yin  2015 | Laparoscopy | YES, 71/75 following CME standards (West grade C, who had intact mesocolon), so 94,67% of the evaluated were valid |
|  | Open | YES, 158/192 following CME standards (West grade C, who had intact mesocolon), so 82,29% of the evaluated were valid |
| Liu  2015 | Laparoscopy | Executed according to principles of CME (clear explanation of procedure), NO explicit report of evaluation of the specimen |
|  | Open |  |
| Gao  2015 | Laparoscopy | YES, 18/18 following CME standards (West grade C), so 100% of the evaluated were valid |
|  | Open | YES, 37/37 following CME standards (West grade C), so 100% of the evaluated were valid |
| Zhao G  2014 | Laparoscopy | Executed according to principles of CME (clear explanation of procedure), NO explicit report of evaluation of the specimen |
|  | Open |  |
| Cong  2014 | Laparoscopy | YES, 42/96 following CME standards (West grade C), 1 of which intramesocolic (not CME), so 97,6% of the evaluated were valid |
|  | Open | YES, 34/82 following CME standards (West grade C), none of which intramesocolic (not CME), so 100% of the evaluated were valid |
| Bae  2014 | Laparoscopy | Executed according to principles of CME (clear explanation of procedure), NO explicit report of evaluation of the specimen |
|  | Open |  |
| Zhao L  2014 | Laparoscopy | Executed according to principles of CME (clear explanation of procedure), NO explicit report of evaluation of the specimen |
|  | Open |  |
| Han  2014 | Laparoscopy | Executed according to principles of CME (clear explanation of procedure), NO explicit report of evaluation of the specimen |
|  | Open |  |
| Guan  2010 | Laparoscopy | Executed according to principles of CME (clear explanation of procedure), NO explicit report of evaluation of the specimen |
|  | Open |  |

**SDC 2b: Characteristics of the studies**

| **Author** | | **Overall post-operative complications**  **(n. patients - %)** | **Complications**  **(Dindo grade III–IV)**  **(n. patients - %)** | **Overall**  **reoperation**  **(n. patients - %)** | **Readmission (n. patients - %)** |
| --- | --- | --- | --- | --- | --- |
| El Nakeeb 2020 | Laparoscopy | NR | NR | NR | NR |
|  | Open | NR | NR | NR | NR |
| Wang  2020 | Laparoscopy | NR | NR | NR | NR |
|  | Open | NR | NR | NR | NR |
| Elbalshy 2019 | Laparoscopy | 1 (13.3%) | NR | NR | NR |
|  | Open | 4 (3.3%) | NR | NR | NR |
| El Fol  2019 | Laparoscopy | 9 (30.0%) | NR | NR | NR |
|  | Open | 8 (26.7%) | NR | NR | NR |
| Jin  2019 | Laparoscopy | 15 (21.1%) | NR | NR | NR |
|  | Open | 20 (24.4%) | NR | NR | NR |
| Pelz  2018 | Laparoscopy | NR | NR | NR | NR |
|  | Open | NR | NR | NR | NR |
| Shin  2018 | Laparoscopy | 125 (18.3%) | NR | NR | NR |
|  | Open | 146 (21.4%) | NR | NR | NR |
| Yu  2018 | Laparoscopy | 25 (23.36%) | NR | NR | NR |
|  | Open | 33 (28.44%) | NR | NR | NR |
| Li  2018 | Laparoscopy | 4 (10.0%) | NR | NR | NR |
|  | Open | 5 (10.4%) | NR | NR | NR |
| Aiypov 2018 | Laparoscopy | NR | NR | NR | NR |
|  | Open | NR | NR | NR | NR |
| Rasulov 2017 | Laparoscopy | NR | NR | NR | NR |
|  | Open | NR | NR | NR | NR |
| Chen  2017 | Laparoscopy | 3 (11,1%) | NR | NR | NR |
|  | Open | 9 (16.36%) | NR | NR | NR |
| Mondal  2017 | Laparoscopy | NR | NR | NR | NR |
|  | Open | NR | NR | NR | NR |
| Huang  2015 | Laparoscopy | 2 (4%) | NR | NR | NR |
|  | Open | 6 (12%) | NR | NR | NR |
| Yin  2015 | Laparoscopy | 9 (12.00%) | NR | NR | NR |
|  | Open | 23 (11.99%) | NR | NR | NR |
| Gao  2015 | Laparoscopy | 3 (16.67%) | NR | NR | NR |
|  | Open | 7 (18.92%) | NR | NR | NR |
| Liu  2015 | Laparoscopy | NR | NR | NR | NR |
|  | Open | NR | NR | NR | NR |
| Zhao G  2014 | Laparoscopy | NR | NR | NR | NR |
|  | Open | NR | NR | NR | NR |
| Cong  2014 | Laparoscopy | 10 (10,4%) | NR | NR | NR |
|  | Open | 11 (13,4%) | NR | NR | NR |
| Bae  2014 | Laparoscopy | 11 (12.9 %) | NR | NR | NR |
|  | Open | 21 (24.7 %) | NR | NR | NR |
| Zhao L  2014 | Laparoscopy | 14 (11.8%) | NR | NR | NR |
|  | Open | 18 (17.6%) | NR | NR | NR |
| Han  2014 | Laparoscopy | 23 (12.99%) | NR | NR | NR |
|  | Open | 33 (22.45%) | NR | NR | NR |
| Guan  2010 | Laparoscopy | NR | NR | NR | NR |
|  | Open | NR | NR | NR | NR |

**SDC 2c:** Oncologic 3-year outcomes

| **Author** | | **Overall recurrence** | **Local recurrence** | **Systemic recurrence** | **Overall**  **survival** | **Disease free**  **survival** |
| --- | --- | --- | --- | --- | --- | --- |
| El Nakeeb 2020 | Laparoscopy | 1 | NR | NR | 71% | NR |
|  | Open | 2 | NR | NR | 74% | NR |
| Wang 2020 | Laparoscopy | 21 | 16 | 5 | 83.75% | 77.50% |
|  | Open | 28 | 25 | 3 | 65.83% | 61.67% |
| Elbalshy 2019 | Laparoscopy | 1 | 1 | 0 | 70.3% | NR |
|  | Open | 2 | 2 | 0 | 73.6% | NR |
| El Fol  2019 | Laparoscopy | 0 | 0 | 0 | 100% | 100% |
|  | Open | 0 | 0 | 0 | 100% | 100% |
| Jin  2019 | Laparoscopy | NR | NR | NR | 87.2% | 83.9% |
|  | Open | NR | NR | NR | 86.0% | 80.5% |
| Yu  2018 | Laparoscopy | NR | NR | NR | 89.81% | NR |
|  | Open | NR | NR | NR | 82.22% | NR |
| Chen  2017 | Laparoscopy | 4 | 4 | 0 | \| 88.1% \| \| --- \| | 84.4% |
|  | Open | 10 | 9 | 1 | 82.9% | 81.3% |
| Huang  2015 | Laparoscopy | 0 | 0 | 0 | 100% | 100% |
|  | Open | 0 | 0 | 0 | 100% | 100% |
| Zhao G  2014 | Laparoscopy | 2 | 2 | 0 | 100% | 91,7% |
|  | Open | 2 | 2 | 0 | 100% | 90,9% |
| Zhao L  2014 | Laparoscopy | NR | NR | NR | 92.0% | 84.6% |
|  | Open | NR | NR | NR | 84.4% | 76.6% |

| **Author** |  | **Overall recurrence** | **Local recurrence** | **Systemic recurrence** | **Overall**  **survival** | **P** | **Disease free**  **survival** | **P** |
| --- | --- | --- | --- | --- | --- | --- | --- | --- |
| Shin  2018 | Laparoscopy | 93 | 9 | 84 | 94.7% | p < 0.001 | 88.7% | p = 0.009 |
|  | Open | 224 | 38 | 186 | 83.7% |  | 82.7% |  |
| Li  2018 | Laparoscopy | 2 | NR | NR | 97.5% | p= 0.67 | NR |  |
|  | Open | 3 | NR | NR | 95.8% |  | NR |  |
| Liu  2015 | Laparoscopy | NR | 2 | NR | 86.36% | p>0.05 | NR | NR |
|  | Open | NR | 1 | NR | 85.00% |  | NR |  |
| Cong  2014 | Laparoscopy | NR | NR | NR | 91,7% | p=0.357 | 94,3% | p=0.823 |
|  | Open | NR | NR | NR | 89,0% |  | 94,6% |  |
| Bae  2014 | Laparoscopy | 10 | 2 | 8 | 77.8% | p= 0.028 | 71.8% | p=0.578 |
|  | Open | 17 | 5 | 12 | 90.3% |  | 83.3% |  |
| Han  2014 | Laparoscopy | 27 | 5 | 22 | 72.5% | p= 0.631^8^ | NR | NR |
|  | Open | 29 | 3 | 26 | 70.37% |  | NR |  |

Oncologic 5-year outcomes
