# Supplementary material for: A global systematic review and meta-analysis on laparoscopic vs open right hemicolectomy with complete mesocolic excision
Source: Int J Colorectal Dis. 2021 Mar 1;36(8):1609–20. doi: 10.1007/s00384-021-03891-0 (PMC8280018; doi:10.1007/s00384-021-03891-0)
Supplement: Supplementary file 3 — (DOCX 24 kb) [file 384_2021_3891_MOESM3_ESM.docx]

SDC 3a: **Risk of bias graph: review authors' judgements about each risk of bias item of RCTs.**


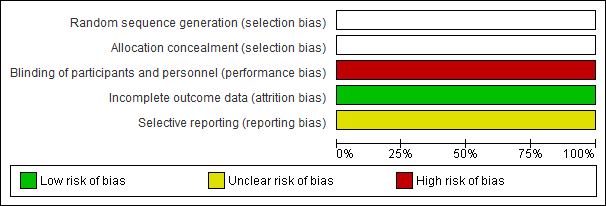


SDC 3b: **Risk of bias summary: review authors' judgements about each risk of bias item for RCTs.**


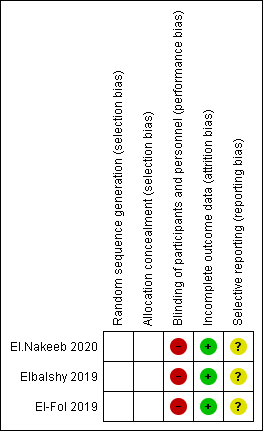


For details on the risk of bias of the included trials see characteristics of the three included RCTs. For an overview of review authors' judgements about each risk of bias item for individual trials and across all trials see Figure 5 and Figure 6

In all RCTs, the Authors do not report any data about random sequence generation and allocation concealment. The blinding of personal was at high risk for the impossibility to perform the blind of the surgeons and nurses.

The Authors do not report a blinding of performance bias/detection bias and of outcome assessment, so these biases were evaluated as unclear risk.

The incomplete outcome data was at low risk, in effect only few patients withdraw.

The Authors do not report the protocol and registration of RCT, for this reason the studies were evaluated as unclear risk
