# Supplementary material for: A global systematic review and meta-analysis on laparoscopic vs open right hemicolectomy with complete mesocolic excision
Source: Int J Colorectal Dis. 2021 Mar 1;36(8):1609–20. doi: 10.1007/s00384-021-03891-0 (PMC8280018; doi:10.1007/s00384-021-03891-0)
Supplement: Supplementary file 4 — (DOCX 27 kb) [file 384_2021_3891_MOESM4_ESM.docx]

**SDC 4. Methodological assessment of the non-randomized studies according to the Methodological Items for Nonrandomized Studies score (MINORS)**

|  | Wang  2020 | Jin  2019 | Yu  2018 | Li 2018 | Shin 2018 | Pelz 2018 | Chen 2017 | Rasulov 2017 | Yin  2015 | Huang 2015 | Liu  2015 | Gao  2015 | Cong 2014 | Bae 2014 | Han 2014 | Zhao L 2014 | Zhao G  2014 | Guan 2010 |
| --- | --- | --- | --- | --- | --- | --- | --- | --- | --- | --- | --- | --- | --- | --- | --- | --- | --- | --- |
| A clearly stated aim | 2 | 2 | 2 | 2 | 2 | 2 | 2 | 2 | 2 | 2 | 2 | 2 | 2 | 2 | 2 | 2 | 2 | 2 |
| Inclusion of consecutive patients | 1 | 1 | 1 | 1 | 2 | 1 | 1 | 2 | 1 | 0 | 0 | 1 | 0 | 0 | 1 | 2 | 1 | 0 |
| Prospective collection of data | 1 | 1 | 1 | 1 | 1 | 1 | 1 | 2 | 1 | 1 | 1 | 1 | 0 | 1 | 1 | 1 | 0 | 0 |
| Endpoints appropriate to the aim of the study (intention to treat) | 2 | 2 | 2 | 2 | 2 | 1 | 2 | 2 | 2 | 2 | 2 | 2 | 2 | 2 | 2 | 2 | 2 | 2 |
| Unbiased assessment of the study endpoint (blinding) | 0 | 0 | 0 | 0 | 0 | 0 | 0 | 0 | 0 | 0 | 1 | 0 | 0 | 0 | 0 | 0 | 0 | 0 |
| Follow-up period appropriate to the aim of the study | 2 | 2 | 2 | 2 | 2 | 2 | 2 | 2 | 1 | 2 | 2 | 2 | 2 | 2 | 2 | 2 | 2 | 2 |
| Loss to follow-up <5% | 2 | 1 | 0 | 0 | 2 | 2 | 2 | 2 | 2 | 2 | 2 | 2 | 2 | 0 | 1 | 1 | 0 | 2 |
| Prospective calculation of the study size | 1 | 1 | 1 | 0 | 2 | 1 | 0 | 0 | 1 | 0 | 1 | 1 | 0 | 2 | 1 | 1 | 0 | 0 |
| A control group having the gold standard intervention | 2 | 2 | 2 | 2 | 2 | 2 | 2 | 2 | 2 | 2 | 2 | 2 | 2 | 2 | 2 | 2 | 2 | 2 |
| Contemporary groups | 2 | 2 | 2 | 2 | 2 | 2 | 2 | 2 | 2 | 2 | 2 | 2 | 2 | 2 | 2 | 2 | 2 | 2 |
| Baseline equivalence of groups | 2 | 2 | 2 | 2 | 2 | 1 | 2 | 2 | 2 | 2 | 2 | 2 | 2 | 2 | 2 | 2 | 2 | 2 |
| Statistical analyses adapted to the study design | 2 | 2 | 2 | 0 | 2 | 0 | 0 | 0 | 1 | 0 | 1 | 0 | 0 | 2 | 2 | 2 | 2 | 0 |
| **Total** | **19** | **18** | **17** | **14** | **21** | **15** | **16** | **18** | **17** | **15** | **18** | **17** | **12** | **17** | **18** | **19** | **15** | **14** |

Item Score: 0 (not reported); 1 (reported but inadequate); 2 (reported and adequate).

The global ideal score being 24 for comparative studies

**Assessment of risk of bias in included studies.** The risk of bias of the included studies was independently assessed by two authors (RC, MF). In order to evaluate the methodological quality of the included studies the methodological index for nonrandomized studies (MINORS) were used. The mean score of the methodological assessment of seven observational studies according to the MINORS was ….. (10-21) (moderate risk).
