# Supplementary material for: A global systematic review and meta-analysis on laparoscopic vs open right hemicolectomy with complete mesocolic excision
Source: Int J Colorectal Dis. 2021 Mar 1;36(8):1609–20. doi: 10.1007/s00384-021-03891-0 (PMC8280018; doi:10.1007/s00384-021-03891-0)

SDC 12. Forest plot of comparison: Laparoscopic versus open CME right hemicolectomy. Intra-operative complications


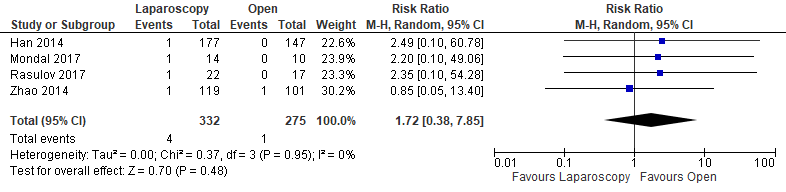

Supplement: Supplementary file 12 — (DOCX 31 kb) [file 384_2021_3891_MOESM12_ESM.docx]
